# Slaying the Swiss Unicorn of Animal Dignity

**DOI:** 10.3390/ani14030507

**Published:** 2024-02-03

**Authors:** David Shaw, Christian Rodriguez Perez, Kirsten Persson

**Affiliations:** 1 Institute for Biomedical Ethics, University of Basel, 4056 Basel, Switzerland; c.rodriguezperez@unibas.ch (C.R.P.); kirsten.persson@unibas.ch (K.P.); 2 Care and Public Health Research Institute, Maastricht University, 6229 ER Maastricht, The Netherlands; 3 Institut für Tierhygiene, Tierschutz und Nutztierethologie, Stiftung Tierärztliche Hochschule Hannover, 30173 Hannover, Germany

**Keywords:** animal ethics, animal dignity, Swiss law

## Abstract

**Simple Summary:**

The Swiss Constitution states that all creatures have dignity, including animals and plants. This paper describes and analyses the Swiss Animal Welfare Act’s approach to animal dignity, and identifies several major problems with the law. In adopting a relative approach to dignity and a very weak test for respecting that dignity, Swiss law enables the exploitation of animals for human benefit, rather than protecting them in line with an absolute concept of dignity. This means that the Act is not compatible with the Constitution, and must be changed.

**Abstract:**

In this article, we describe and analyse the Swiss legislation relating to animal dignity. We conclude that previous criticisms of the law do not go far enough: far from protecting animal dignity, the Swiss law not only undermines such dignity but itself serves as a means to ensure that animals can be used merely as a means, and not treated with respect. As such, the Swiss Animal Welfare Act is deeply unethical and undermines the constitutional requirement to treat animals with dignity.

## 1. Introduction

The concept of dignity has a long and somewhat troubled history in bioethics. Although dignity is often used in bioethical discourse and is referred to in many legal instruments such as the UN Declaration on Human Rights [1], the term remains somewhat amorphous [2]. Generally, it is used to refer to the inherent worth of human persons, often in connection with the idea that people should be treated with respect, and specifically with the Kantian rule that people should never be used merely as a means but treated as ends in themselves [3]. These are all valuable concepts, but the phrase dignity itself has been strongly criticised for not adding any conceptual value to bioethical discourse, except as shorthand for other concepts. In her oft-cited article on this topic, Ruth Macklin famously claimed two decades ago that “dignity is a useless concept”, and specifically that “appeals to dignity are either vague restatements of other, more precise, notions or mere slogans that add nothing to an understanding of the topic” [4] (We provide more background on the contested concept of dignity in the next section of this paper).

Another issue affecting the concept of human dignity is that it is often wielded by people with religious affiliations who wish to defend theological positions without specific recourse to theological arguments [5], though the nature of dignity means that counter-arguments are readily available. For example, opponents of euthanasia often argue that helping someone die violates their human dignity; equally, however, proponents of assisted dying often argue that it is against human dignity to deny someone access to euthanasia.

Nonetheless, as already mentioned, dignity continues to play a prominent role in bioethical discourse, even if it does not have much philosophical heft of its own. In Switzerland, where politicians regularly claim that animals enjoy some of the best protections anywhere in the world [6], the concept of dignity has even been enshrined in the law regarding animals. This might come as a surprise to some proponents of human dignity, who also use the concept to differentiate humans from other, supposedly lesser creatures. (One of the arguments against the creation of hybrid embryos containing human and animal DNA was that it violated human dignity [7].) At first glance, it might be assumed that enshrining animal dignity in Swiss legislation is an important step towards greater protection for non-human animals.

In this article we explore how dignity is described in the Swiss law, and add to already existing criticisms of this legislation. The main problems with the current law are that, by using a relative concept of dignity it does not protect (any) animals in any meaningful sense; the test in the Swiss law for non-instrumentalisation actually serves to facilitate instrumentalisation, and this in turn means that a law designed to protect animal dignity itself facilitates exploitation of animals.

## 2. The Concept of Dignity

The term “dignity” is used ambiguously within and between law, theology, and philosophy [8] and originally refers to “human dignity”. Debes [8] defines four categories of meaning for (human) “dignity”:

1. In terms of gravitas: a graceful attitude and behaviour, including, e.g., an elegant way of speaking or the ability to save face when being insulted.
2. In terms of integrity: to conform to personal or societal normative standards of character and behaviour.
3. In terms of status: a noble/elevated social position.
4. In terms of inherent worth: an unconditional worth that is shared by all humans.

For ethics, the second and fourth categories are most relevant. In Kantian philosophy, for example, “dignity” is used to refer to the inherent worth of human persons, often in connection with the idea that people should be treated with respect, and specifically with the Kantian rule that people should never be used merely as a means but treated as ends in themselves [3]. For a comparison with the notion of animal dignity, it is important to emphasise that human dignity as an inherent worth in the Kantiant sense is, thereby, unconditional, unearned, and carried by all humans qua being human.

Additionally, as Praetorius [9] emphasises, human dignity in its manifold definitions is often explicitly understood as a criterion for the demarcation of human beings from the rest of the (living) world, while, at the same time, human dignity serves as a blueprint for the concept of animal dignity (or the “dignity of creature”, which can for the purpose of this chapter be understood as a synonym). This ambiguity obviously creates a field of tension for defining and understanding animal dignity. For Praetorius, who discusses several potential notions of “dignity of creature”, the only way to meaningfully define and apply animal dignity must be based in a change of “theoretical and ethical attitude towards the world” (p. 43, translated by the authors) because humans cannot grasp the essence of the non-human living world, and must therefore respect it by not disposing of it. Against the background of Praetorius’ approach, animal dignity as it is defined in the Swiss law is revealed as only a very first step towards this far-reaching change in awareness.

## 3. The Swiss Law

In 1992, Switzerland introduced the first legislation in the world designed to protect animal dignity. In fact, this was not merely legislation, but a new constitutional principle. However, it was not motivated specifically by a desire to protect non-human animals, but more generally targeted at limiting the exploitation of reproductive and genetic material from non-human lifeforms, leading to the addition to the Constitution of the need to respect the “dignity of living beings” [10]. Indeed, while it is quite widely known that the Swiss Constitution protects the dignity of animals, it is less well known that article 120 focuses not on dignity but on “non-human gene technology”. Its precise wording is: “The Confederation shall legislate on the use of reproductive and genetic material from animals, plants and other organisms. In doing so, it shall take account of the dignity of living beings as well as the safety of human beings, animals and the environment, and shall protect the genetic diversity of animal and plant species” [11].

This origin of the concept of animal dignity is rather unfortunate. Rather than extending the idea of human dignity to non-human animals, other creatures were included along with plants and trees and all other biological lifeforms. Such inclusion of the broader non-human living world is not the change in “theoretical and ethical attitude towards the world” that Praetorius suggests (see above), but rather reinforces the idea that humans can define the level of protection these beings deserve while they are used. Of course, all life deserves a certain level of protection, just as some non-living environmental features do. However, suggesting that daffodils and grass have dignity rather undermines the claim that animals do; it simultaneously elevates animals’ moral status while demoting them to the level of leaves. Plants deserve protection, but using dignity as the concept to protect them seems misguided, even if it could be argued for. Of course, the same could be said of animal dignity (and as we have seen, even human dignity), but if we wish to protect animals, then extending the concept used to protect them to plants at the same time is perhaps not the best start. Here, already, the concept of dignity appears to be being used in a different sense from human dignity, a divergence which will prove very problematic. As argued previously, “The profound distinction between this and the concept of human dignity presents a potential challenge to folk intuition: it might be difficult to understand a familiar concept in a second, fundamentally different way” [12]. As we will show below, this distinction is very much in line with the “utilitarianism for animals, Kantianism for people” position introduced by Robert Nozick in 1974, which proves useful for the continued instrumentalisation of animals by humans [13].

In 2008, the Animal Welfare Act was introduced, implementing the Constitution’s dignity concept in practice. The first article of the Act states that “The purpose of this Act is to protect the dignity and welfare of animals”—an auspicious opening [14]. However, Article 2 states that “The present Act applies to vertebrates”; thus, the Act provides only limited protection for invertebrates. Specifically, Article 2 continues “The Federal Council decides to which invertebrates it applies and to what extent” [14]. So, in the space of three sentences we have moved from protecting (all) animals to protecting vertebrates to also protecting some invertebrates. How does the Council decide which invertebrates to protect? “In doing so, it is guided by scientific knowledge on the sentience of invertebrate animals”. Therefore, the Act does not even seek to protect the dignity of all animals; it seeks to protect only the dignity of vertebrate and some sentient invertebrate animals.

Article 3 of the Act defines animal dignity. In fact, the definition of animal dignity in the Act conveniently also provides the test for when dignity is violated:

“Dignity means the inherent worth of the animal that must be respected when dealing with it. If any strain imposed on the animal cannot be justified by overriding interests, this constitutes a disregard for the animal’s dignity. Strain is deemed to be present in particular if pain, suffering or harm is inflicted on the animal, if it is exposed to anxiety or humiliation, if there is major interference with its appearance or its abilities or if it is excessively instrumentalized” [14].

This is an interesting notion of dignity. With its reference to inherent worth, the first sentence closely resembles the concept of human dignity and is consistent with the above-mentioned fourth category of Debes relating to Kantianism. The second sentence throws any such concept of dignity out of the window with the claim that imposing strain in the absence of justifying (human) interests violates the creature’s dignity. According to Kunzmann [15], animal dignity is understood in different ways, two of which are reflected in this article. Animal dignity is either attributed to the animal as an inherent worth (or some species-specific properties) which is to be respected, mirrored in the first sentence of the act, or, animal dignity is attributed to the human action (or attitude) in a sense that by performing an action animal dignity is preserved. The latter understanding is expressed in the description of the weighing process in the second part of the act.

The weighing of human interests and animal instrumentalisation implies, of course, that there is a certain level of instrumentalisation which does not violate animal dignity—in stark contrast to the concept of human dignity. This clearly shows that we are far from the change of “theoretical and ethical attitude towards the world” suggested by Praetorius. Furthermore, in humans, the test for instrumentalisation is whether you are using a person merely as a means (see below). In animals the test shifts from whether you are using the animal merely as a means to whether the end justifies the means. Let us now explore these issues in more depth.

### 3.1. Dignity as a Relative Concept

This is the clear central tension in the Swiss law. As Bollinger states, “The Swiss dignity protection concept is based on the conviction that animals exist—and have to be protected by law—for their own sake, not primarily for human interests” [10]. This is an important concept, and it is indeed vital that it is included in the constitution. However, as Bollinger also states, “in contrast to human dignity, animal dignity is only given a relative value in Swiss law, meaning that violations of animal dignity usually can be balanced and legally justified by prevailing human interests” [8]. This point was also made by the Swiss Academy of Medical Sciences and the Swiss Academy of Sciences’ Ethics Committee for Animal Experimentation in 2010 [16].

This is a new and different type of dignity. The whole point of dignity as a concept is that it cannot be relative—dignity is either respected or it is not. (Bollinger goes on to criticise instances where the test is not being properly applied in Switzerland, but the test itself is clearly flawed.) In humans, one simply cannot justify exploitation or instrumentalisation of people by saying that you are using them for something more important than their dignity. The test for respecting human dignity is whether someone is being instrumentalised, not whether the thing you are instrumentalising them for is important enough. The latter test, and the one in the Swiss law, is a utilitarian test, which is entirely inappropriate to any true concept of dignity and rather uses the same old Nozickean “utilitarianism for animals, Kantianism for people”.

It can even be argued that, in institutionalising a relative concept of animal dignity, the Swiss law directly challenges the implied absolute concept of dignity in the constitution, and as such may itself be unconstitutional. Indeed, even if the constitutional concept of dignity is interpreted as being only a relative concept, the way in which the test for respecting animal dignity can itself be used to undermine (even relative) dignity could be seen as unconstitutional (see below).

### 3.2. The “Merely” Test for Respecting Dignity in Switzerland

Of course, in humans, the Kantian injunction is not simply to avoid using people as a means—it is to not use them merely as a means. In contrast, using animals in a way that is in accordance with their dignity in terms of Swiss law basically means that you can use them as a means as long as it is not merely as a means—and all you need to do to meet the minimum test for “merely” is check whether using them as an end is important enough for you and your aims. Imagine if the “merely” check for humans was whether the aim you want to exploit them for is important enough to use them merely as a means. That would be no protection of dignity at all, and that is precisely what vertebrates and other animals that are perceived as sentient in Switzerland enjoy.

### 3.3. Using the Law on Animal Dignity to Instrumentalise Animals

However, in fact, it is not just that the Swiss law simultaneously says animals have dignity while they are not treated that way in reality. It is not even that the Swiss law says they have dignity while other aspects of the law show that they do not really. It is even worse than that, because the concept animal dignity in Swiss law is itself merely a means of instrumentalising them. Prior to the introduction of the Animal Welfare Act, non-human creatures in Switzerland at least enjoyed the theoretical protection afforded them by the Constitutional amendment in 1992. However, the Act not only fails to implement those protections in practice, and not only undermines the Constitution, but itself provides a means of instrumentalising animals—and ironically, that means is the test for respecting animal dignity itself. Indeed, the test for respecting animal dignity while still instrumentalising animals is to provide some basic justification for that instrumentalisation. However, the threshold is so low (such that it is really a non-test), that the very test becomes a tool for anyone who seeks to instrumentalise them in a “legitimate” way.

### 3.4. Implications of the Concept of Absolute Dignity on Other Aspects of the Swiss Law: Killing, Location, Speciesism and Humiliation

If animal dignity were more meaningfully understood as absolute rather than relative, further aspects of the Swiss animal welfare legislation would need to be questioned, as there are also other quirks in the law.

As Bollinger points out, the dignity protections in the Act do not extend to protection of life, which is one of the most important human rights [10]. If this protection were provided, one might imagine that people would no longer (for example) be able to kill animals for meat in Switzerland. A similar situation can be observed in Germany, where animal life is protected by the law and a good reason is demanded for causing harm (including death) to animals (§1 German Animal Welfare Act). Killing animals for food purposes is, however, considered to be compatible with the German Animal Welfare Act. Thus, if animals’ lives were protected by the concept of dignity in the Swiss law, it would not make any difference as our overriding interest in eating them (if deemed by us to be important enough) would outweigh any concerns about their dignity. Indeed, the threshold in the law is so low that consumers’ overriding interest in being able to obtain a one-franc hot dog at Ikea, is apparently in line with respecting their dignity despite knowing all the suffering that this sort of production implies for animals [17,18] [The Ikea hotdog is an iconic product. In Switzerland today, it costs CHF 1.50 and contains chicken meat coming from the Swiss farming industry. As discussed, while Switzerland is often described as having one of the strictest animal welfare regulations worldwide [6], that does not change the fact that chickens aimed at cheap products tend to live miserable lives].

Furthermore, another issue with the way dignity is misapplied to animals in Switzerland is that their level of protection varies dependent on their location or their “function” from a human perspective. For example, a rabbit in a private home benefits from different laws from those that apply to a similar rabbit in animal research [19]. This fundamental legal flaw has been addressed previously. Treating animals differently depending on the way humans mean to use them represents a context-dependent, extrinsically defined protection of animals, in contrast to the inherent value that is claimed to be legally attributed and should be context-independent [20]. Given that dignity is meant to be absolute, having different levels of protection for creatures with the same innate dignity makes no sense—unless the same being can have two (relatively) different dignities, which again makes no sense.

A related point is that, as well as the aforementioned discrimination against some invertebrates, speciesism is also evident in the Swiss legislation; animal species used as pets have greater dignity than species used as cattle. To give a specific example: “The import, transit and export of cat and dog pelts and products made from them is forbidden, as is trade in pelts and products of this kind [14]. This clause estimates that the killing techniques of these cats and dogs in other countries are inhumane, but in the case of dogs it is basically the same technique used in Switzerland for cattle [21].

Another odd aspect is that animals must not be “humiliated” as doing so violates their dignity. One example of humiliation is using animal organs for transplantation into humans [10]—a lifesaving medical procedure that unfortunately leads to the death of one animal. In contrast, however, the nationwide consumption of animals for their meat is apparently not humiliating even though it is not necessary or lifesaving. This is perhaps the perfect example of just how relative the Swiss approach to animal dignity is: an animal cannot be killed to save a life, because that would be contrary to dignity; but the same animal can be killed and slaughtered and eaten because people like the taste. At the very least, this is indicative of a very confused legislative framework.

### 3.5. Implications of the Swiss Approach to Animal Dignity

The Swiss law on animal welfare is deeply conflicted. The Constitution says that all animals (and plants) have dignity; the law states that they have moral value regardless of sentience while restricting any protections to vertebrates and certain sentient invertebrates, but any such protections can be used regardless of dignity because human interests prevail at every turn. In fact, and as might be expected given previous criticisms of the nebulous nature of the concept of dignity, it appears that the word in this context really signifies “value” rather than dignity—and human values trump animal values. It is unfortunate that Swiss politicians frequently claim that Switzerland has some of the best animal protections in the world when they are really so weak; indeed, this claim is often used to argue against further protection of animals, so in that sense the Animal Welfare Act is ironically (and somewhat hypocritically) itself being used to justify no further protections for animals.

Ultimately, the principal animal dignity “protection” in the Swiss Animal Welfare Act is actually merely a means of instrumentalising them. This is not only contrary to the concept of animal dignity in the constitution, but also contrary to human dignity, for two reasons. First, by claiming to protect animal dignity while actually seeking to undermine it, the law itself engages in undignified underhand tactics. Second, by eroding the already questionable notion of animal dignity further by means of a utilitarian test for a deontological rule, the Act could lead to violation of human dignity if the Act’s test were to be exported and used in justification of exploitation of humans.

For all these reasons, changes must be made to the Swiss law. If the constitutional requirement to treat all creatures with dignity is to be taken seriously, then the Animal Welfare Act must abandon its relative conception of dignity and move to an absolute concept of dignity. This would entail substantial consequences; for example, that animals could not be killed to be eaten. If this option proves unpalatable, the law could instead adopt an alternative concept to convey the moral worth of animals—perhaps “integrity” as we have suggested elsewhere [12] and as it can be found, for example, in the Dutch Animal Law [22] (“Wet dieren”, Art. 1.3.) explaining the meaning of “intrinsic value” (“intrinsieke waarde”) of animals. However. this would in turn require dropping the reference to dignity in the constitution.

## 4. Conclusions

Ultimately, we have concluded that the Swiss approach to animal dignity is fundamentally flawed for at least three reasons. First, it confers an inferior, relative concept of dignity on animals rather than the absolute concept that applies to humans. This is in conflict with the Constitution, which implies equal dignity for all living creatures. This use of a relative concept of dignity also causes further inconsistencies in the law, with animals being given no protection from killing, and being accorded greater or lesser levels protection depending on their location or species.

Second, the test to check whether a proposed course of action is compatible with animal dignity is fundamentally flawed, because the only check is whether the human ends justify using the animal as a means. This means that the law provides no meaningful protection to any animals.

Worse, (and third,) this flaw in the test in the law itself subordinates animal interests to human interests, and thus facilitates the instrumentalisation of animals rather than protecting it. Thus, the law not only provides no meaningful protection but it enables violations of animal dignity and may well be unconstitutional as a result.

In addition, these three fundamental flaws also serve to devalue human dignity in a twofold sense; because it is undignified to claim to defend other’s dignity while actually undermining it, and because the weak instrumentalisation test could itself erode our respect for humans.

For all its flaws, at least the concept of human dignity serves as a theoretical foundation for fundamental human rights. In Switzerland at least, the concept of animal dignity actually not only sets back the cause of protecting animals from exploitation by humans, but actively facilitates it. Therefore, the terminology used in Swiss legislation and the Constitution should be changed (perhaps to “integrity”, as we have suggested elsewhere [10])—the only alternative is for Swiss law to actually treat animals in Switzerland with absolute dignity, in accordance with the country’s Constitution. Of course, doing that would involve significant societal change at a level that many humans may be unwilling to accept.

## Data Availability

Data are contained within the article.
